# A Combined Transcriptomic and Machine Learning Study Reveals PAX8 as a Promising Diagnostic Biomarker in Endometriosis

**DOI:** 10.1155/humu/9501547

**Published:** 2026-04-17

**Authors:** Xiaoli Zhu, Li Zhong, Yanlin Xu, Yuanxia Zou, Manyun Liu, Xiaoqian Tong

**Affiliations:** ^1^ Department of Gynaecology and Obstetrics, Yingtan 184 Hospital, China RongTong Medical Healthcare Group Co. Ltd., Yingtan, Jiangxi, China; ^2^ Department of Gynaecology and Obstetrics, Shangrao People′s Hospital, Shangrao, Jiangxi, China; ^3^ Department of Obstetrics and Gynecology, The First Affiliated Hospital of Changsha Medical University, Changsha, Hunan, China

**Keywords:** biomarker, endometriosis, machine learning

## Abstract

**Background:**

Endometriosis (EM) is a chronic, estrogen‐dependent disease that lacks reliable noninvasive diagnostic biomarkers. This study was aimed at evaluating the diagnostic value of PAX8 using integrated transcriptomic and machine learning analyses.

**Methods:**

Transcriptomic data from the GSE141549 dataset were analyzed to identify differentially expressed genes (DEGs). Weighted gene coexpression network analysis (WGCNA), immune infiltration profiling, and functional enrichment (GO, KEGG, and GSEA) were conducted. Random forest (RF), support vector machine (SVM), and logistic regression (LR) models were trained and validated through five‐fold cross‐validation.

**Results:**

A total of 887 DEGs were identified, among which PAX8 was significantly downregulated in ectopic tissues and identified as a key diagnostic feature by both RF and WGCNA. GSEA revealed that PAX8‐related gene sets were enriched in biological processes such as cilium organization and wound healing, and KEGG analysis indicated involvement in calcium signaling, JAK‐STAT signaling, and focal adhesion pathways. Immune infiltration analysis further supported an immunomodulatory role of PAX8.

## 1. Introduction

Endometriosis (EM) is a common estrogen‐dependent inflammatory condition defined by the presence of endometrial‐like tissue outside the uterus. It affects approximately 10% of women of reproductive age globally and is associated with symptoms such as chronic pelvic pain, dysmenorrhea, difficulties with sexual intercourse, and infertility [1, 2]. These symptoms have a serious impact on quality of life and reproductive health [3]. Although histologically confirmed laparoscopy remains the gold standard for diagnosis, its invasiveness and high price make it unsuitable for population‐level screening [4]. Noninvasive diagnostic tools, such as imaging or serum markers, such as CA125, lack the sensitivity (SEN) and specificity (SPE) required to detect subtle or deep lesions [5]. Therefore, the discovery of new biomarkers with higher diagnostic accuracy (ACC) and a minimal or noninvasive nature is urgently needed.

Due to the limitations of current diagnostic modalities, the search for reliable, noninvasive molecular biomarkers is an increasingly important endeavor in EM research. EM lesions exhibit tumor‐like features such as invasion, angiogenesis, and recurrence, suggesting that molecular features may have diagnostic and prognostic value [1]. However, many candidate markers lack consistency across tissue types and patient situations, especially in hormone‐treated or fibrotic lesions [6]. Therefore, it is crucial to identify stable markers with high diagnostic ACC in different pathological contexts.

In recent years, machine learning and deep learning techniques have been increasingly adopted in disease classification and diagnosis, including brain tumor prediction based on MRI imaging. These approaches demonstrate the feasibility and power of computational modeling in improving diagnostic performance across diseases. Paired box gene 8 (PAX8) is a transcription factor that plays an important role in Müllerian phylogeny and is stably expressed in gynecological tissues such as the endometrium and ovary [7]. PAX8 still shows strong and consistent immunoreactivity in fibrotic or hormone‐treated tissues compared to traditional markers such as CD10, estrogen receptor (ER), and progesterone receptor (PR) [7]. Furthermore, PAX8 has been identified as a highly sensitive epithelial marker for extragenital EM, with expression maintained even under hormonal therapy, highlighting its potential utility in detecting ectopic endometrial epithelium [8]. Although previous studies have reported the expression of PAX8 in endometriotic tissues based on immunohistochemical analyses, there is still a lack of systematic evaluation of its diagnostic potential using large‐scale transcriptomic data, particularly within a machine learning–based analytical framework. Building upon prior work, this study integrates differential expression analysis, immune infiltration profiling, and multiple machine learning algorithms to preliminarily investigate the potential clinical utility of PAX8 as a diagnostic biomarker. By incorporating the immune microenvironment context and data‐driven modeling strategies, we aim to provide a reference for addressing current challenges related to biomarker stability and translational applicability.

In this study, we analyzed transcriptomic data from the GEO dataset GSE141549, comprising 224 peritoneal tissue samples, including 85 from patients with EM and 139 from healthy controls. Differentially expressed genes (DEGs) were identified using the limma package, followed by Gene Ontology (GO) and Kyoto Encyclopedia of Genes and Genomes (KEGG) enrichment analyses. Among the significantly downregulated genes, PAX8 emerged as a key candidate involved in developmental pathways relevant to endometrial biology. To assess its diagnostic potential, we applied multiple machine learning algorithms—random forest (RF), support vector machine (SVM), and logistic regression (LR)—for sample classification, with PAX8 demonstrating strong discriminative performance. Additionally, integration with weighted gene coexpression network analysis (WGCNA) and immune infiltration profiling further substantiated its potential as a clinically relevant biomarker. Collectively, these findings provide a robust foundation for the future application of PAX8 in the early, noninvasive molecular diagnosis of EM.

## 2. Materials and Methods

### 2.1. Data Collection and Processing

In total, 224 endometrial samples were included in this study, comprising 85 deep‐infiltrating endometriosis (DiE) samples and 139 non‐DiE control samples. The DiE group consisted of ectopic endometrial tissues from patients with pathologically confirmed DiE, while the control group comprised eutopic endometrial tissues from patients without EM who underwent surgery for benign gynecological conditions (e.g., uterine fibroids and ovarian cysts).

**Figure 1:**
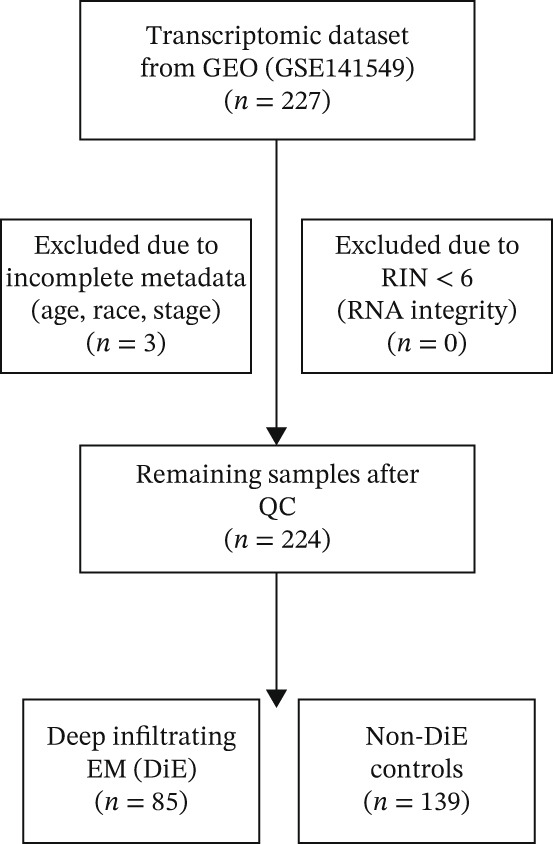
The flowchart depicts the inclusion and exclusion process in the study.

In this study, we utilized the high‐throughput transcriptomic dataset GSE141549 from the GEO database, which covered a variety of sample types, including eutopic endometrium, peritoneal tissue, and different types of endometriotic lesions. The samples we used are from 85 DiE lesion patients and 142 non‐DiE patients [9]. Gene expression profiling in this dataset was performed using two microarray platforms: the Illumina HumanHT‐12V4.0 Expression BeadChip (Platform ID: GPL10558) and the Illumina HumanWG‐6V2.0 Expression BeadChip (Platform ID: GPL13376). These platforms were designed for high‐throughput gene quantification and whole‐genome coverage, respectively, catering to comprehensive transcriptomic analysis. Raw data were preprocessed through standard normalization procedures, and probe identifiers were mapped to corresponding gene expression matrices, providing a robust foundation for subsequent differential expression analysis and integrative multiomics studies. Raw gene expression profiles (CEL files) were downloaded from the GEO database under accession GSE141549 and imported into R (v4.3.2). Samples lacking complete clinical metadata (age, race, and disease stage) or with RIN < 6 were excluded. After exclusion, 224 samples (85 DiE; 139 non‐DiE controls) remained for downstream analysis. Probes were reannotated and collapsed to unique Entrez IDs using http://org.Hs.eg.db (v3.17). Quantile normalization was applied with the limma package to correct for technical variation, followed by ComBat (sva v3.48.0) to remove platform‐specific batch effects (GPL10558 vs. GPL13376). Hierarchical clustering on Euclidean distances identified three outlier samples that were subsequently removed. Postnormalization principal component analysis (PCA) confirmed clear separation between DiE and control groups (Figure 1). The final expression matrix was log2‐transformed and *z*‐score standardized across genes prior to differential expression and machine learning analyses. After quality control and batch‐effect correction, a total of 224 samples from the GSE141549 dataset were included in the final analysis, comprising 85 DiE lesion samples and 139 non‐DiE tissue samples. All downstream analyses were consistently conducted by comparing DiE samples with non‐DiE samples.

### 2.2. Identification of DEGs in the EM

To identify DEGs with significant expression differences between DiE samples and non‐DiE samples, we first preprocessed the gene expression matrix to ensure sample quality and consistency. DEG analysis was performed using the “limma” package in R, which employs linear modeling combined with empirical Bayes moderation to improve variance estimation and enhance the robustness and reliability of the results. The primary comparison was conducted between DiE and non‐DiE tissue samples. Genes were considered DEGs if they exhibited an absolute log2 fold change (|log2*F* *C*|) > 1 and a Benjamini–Hochberg false discovery rate (FDR) adjusted *p* value < 0.05, corrected using the method.

### 2.3. GO and KEGG Enrichment Analyses of DiE DEGs

To explore the biological processes (BPs) and molecular mechanisms associated with DEGs, we performed GO and KEGG pathway enrichment analyses. DEGs were first converted from gene symbols to Entrez gene IDs using the org.Hs.eg.db annotation database, and genes lacking corresponding Entrez IDs were excluded to ensure data integrity. GO enrichment analysis was conducted using the enrichGO function in the “clusterProfiler” R package, with the ontology categories set to “all,” encompassing BP, molecular function (MF), and cellular component (CC). Significance thresholds were defined as a *p* value < 0.05 and an FDR‐adjusted *q* − value < 0.05. For KEGG pathway enrichments, we used the enrichKEGG function, specifying the organism as *Homo sapiens* (hsa) and setting the key type to “kegg” using the same cutoffs as the enrichGO function. Both GO and KEGG results were visualized using barplots and dotplots to highlight the most significantly enriched terms and pathways. All analyses were conducted in R, with parallel computing enabled through the doParallel package to improve efficiency, and visualizations were generated using the enrichplot, ggplot2, and GOplot packages.

### 2.4. Identification of EM‐Specific Genes Using Machine Learning Methods

To further investigate diagnostic markers that are associated with EM, three machine learning methods (SVM, RF, and LR) were constructed based on DEGs for the classification of DiE and non‐DiE samples. To enhance the generalizability and prediction performance of our models, we employed a five‐fold cross‐validation strategy for systematic training and used ACC to adjust the hyperparameters of the models. Specifically, the entire dataset was randomly divided into five subsets in a 4:1 ratio, ensuring that each fold maintained a balanced distribution of EM and healthy samples. This stratified partitioning minimized potential biases due to class imbalance. Model hyperparameters were tuned using Optuna [10] based on the ACC value. For each model, we employed a systematic search space to explore suitable parameters that would yield optimal classification performance. In this study, SVM was optimized for regularization strength (*C*), kernel, gamma, and degree. The RF was tuned for the number of trees, max tree depth, split criteria, and feature selection strategy. And the LR model was optimized for regularization strength, solver type, and number of iterations. The detailed hyperparameter research spaces for each model are shown in Table 1.

**Table 1:** The details of the hyperparameters in the three models.

Model	Hyperparameter	Range
SVM	*C*	1e − 3 to 1e3
	Kernel	Linear, rbf, poly, and sigmoid
	Gamma	Scale and auto
	Degree	2~5 (for “poly” kernel)

RF	n_estimators	100~1000
	max_depth	3~20
	min_samples_split	2~20
	min_samples_leaf	1~10
	max_features	sqrt and log2
	Bootstrap	True and false
	Criterion	Gini, entropy, and log_loss

LR	*C*	0.01~100
	Solver	Lbfgs and liblinear
	max_iter	100~2000

### 2.5. Construction of the Coexpression Network and Hub Module WGCNA

To identify gene modules exhibiting coordinated expression patterns in EM, the WGCNA was performed using the “WGCNA” R package. To ensure data quality, the “goodSampleGenes” function was first applied to detect and remove outlier samples or genes with excessive missing values. Subsequently, an appropriate soft‐thresholding power (*β*) was selected to ensure a scale‐free topology of the network, and a weighted adjacency matrix was constructed. This matrix was then transformed into a topological overlap matrix (TOM) to measure network interconnectedness. Genes were hierarchically clustered based on TOM‐based dissimilarity, and modules were identified using a dynamic tree cut algorithm with a minimum module size of 30. Module eigengenes (MEs) were calculated to summarize the expression profiles of each module, and the relationships between modules and external clinical traits were evaluated by computing the Spearman correlation coefficients. Modules showing the highest correlation with the phenotype of interest were considered as key modules for further analysis.

### 2.6. Gene Set Enrichment Analysis (GSEA) of Coexpressed Gene Sets for Signature Genes

To investigate the potential biological functions associated with signature genes, we performed GSEA. For each target gene, samples were divided into high‐ and low‐expression groups according to the median expression levels. We then calculated the average expression values for all other genes in the two groups and derived the log2FC between them again to obtain a ranked gene list. The ranked list was subjected to GSEA using the clusterProfiler R package, with gene sets obtained from the Molecular Signatures Database (MSigDB, Version 2024.1), including GO (c5) and KEGG (c2) gene sets. The enrichment analysis was performed using default permutation settings, and results with a nominal *p* value < 0.05 were considered statistically significant. Finally, the top‐enriched terms from both the high‐ and low‐expression groups were visualized using the enrichplot package to illustrate the underlying BPs and pathways associated with differential gene expression.

### 2.7. Immune Analysis of Signature Genes

To systematically characterize the immune microenvironment associated with EM and explore its potential relationship with key regulatory genes, we performed immune cell deconvolution using the CIBERSORT algorithm to estimate the relative proportions of 22 immune cell types in each sample using the LM22 signature matrix. The normalized gene expression matrix was analyzed using the LM22 signature matrix, which enables the estimation of 22 distinct human immune cell subsets, including T cells, B cells, macrophages, dendritic cells, and natural killer (NK) cells. The algorithm was executed with 1000 permutations to ensure statistical robustness. Only samples with a CIBERSORT *p* value < 0.05 were retained for further analysis, indicating reliable immune composition estimates. We compared the relative abundances of immune cell types between EM and control groups using the Wilcoxon rank‐sum test, followed by multiple testing correction using the Benjamini–Hochberg method. Visualization of immune infiltration patterns was carried out using bar plots and violin plots, allowing us to explore immune differences across conditions and identify immune cell types potentially associated with the pathogenesis of EM.

## 3. Results

### 3.1. Data Processing

The dataset used in this study included 227 endometrial samples from the GEO database under the accession number GSE141549. To ensure the reliability and usability of the data, we first conducted hierarchical clustering analysis on all samples. Three clear outlier samples exhibited substantial deviations in global expression patterns compared to the rest of the cohort (Figure 2a). To avoid potential biases in downstream analyses, these outliers were removed, resulting in a final dataset of 224 samples for further investigation.

Figure 2The clustering and PCA of DiE and non‐DiE samples. (a) The hierarchical clustering analysis of the samples. (b) PCA of the samples.

**Figure:**
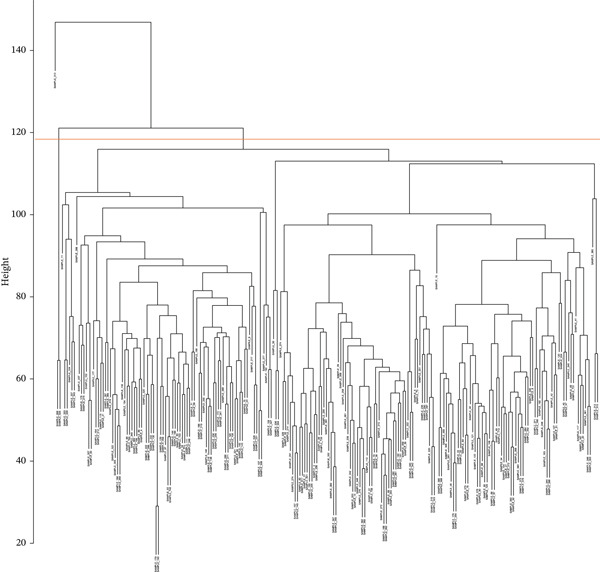
(a)

**Figure:**
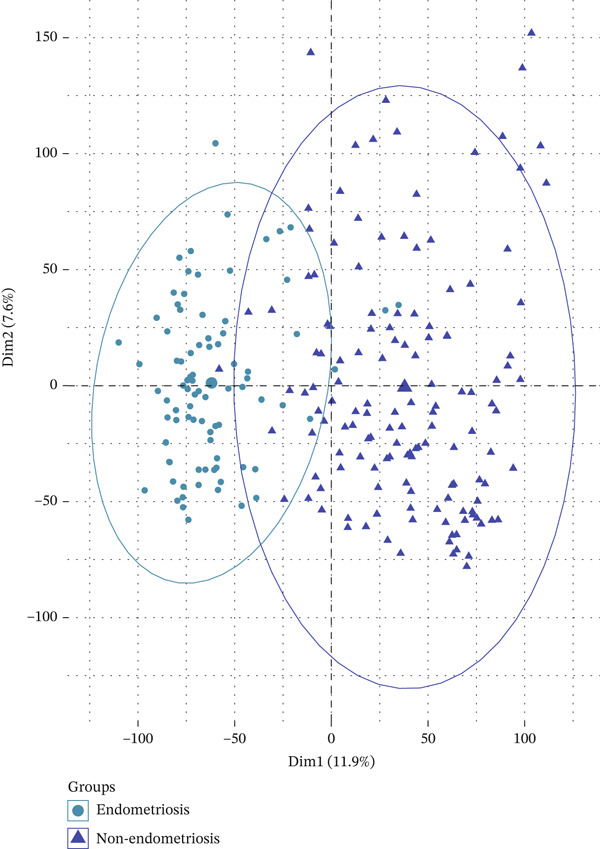
(b)

Among the retained samples, 85 were diagnosed with EM, while the remaining 139 samples served as nonendometriotic controls. All expression data underwent normalization to eliminate technical variation and enhance comparability. Subsequently, we performed PCA to evaluate the overall distribution and separability of the samples in the dataset (Figure 2b). The PCA results revealed a distinct trend of separation between EM and control samples in the principal component space, suggesting potential transcriptional differences between the two groups.

### 3.2. Identification and Analysis of DEGs

In the GSE141549 dataset, 887 DEGs were identified between 85 DiE samples and 139 non‐DiE samples by using differential expression analysis. Among the identified DEGs, 548 genes were upregulated and 339 were downregulated according to the screening criteria of |log2*F* *C*| > 1 and *p* value < 0.05. The overall distribution of expression changes is visually represented in the volcano plot (Figure 3a). To further investigate the expression profiles of these DEGs, we selected the Top 50 upregulated and Top 50 downregulated genes for hierarchical clustering and heatmap visualization. As shown in Figure 3b, these genes displayed clear differential expression patterns between the DiE group and the non‐DiE group, indicating the potential biological relevance of these genes in the context of disease progression and pathogenesis.

Figure 3Differential expression analysis in DiE and non‐DiE. (a) Volcano plots illustrating DEGs between DiE and non‐DiE. (b) Heatmap of gene expression different between DiE and non‐DiE.

**Figure:**
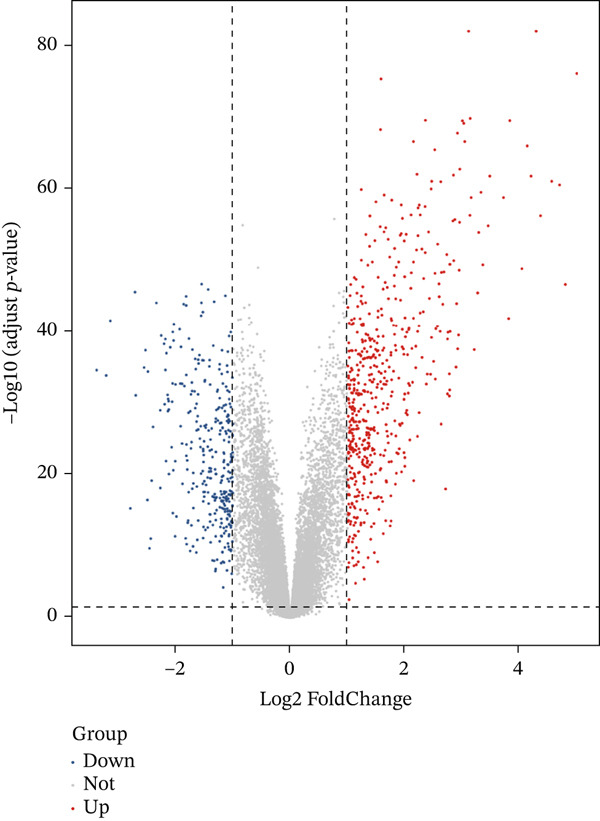
(a)

**Figure:**
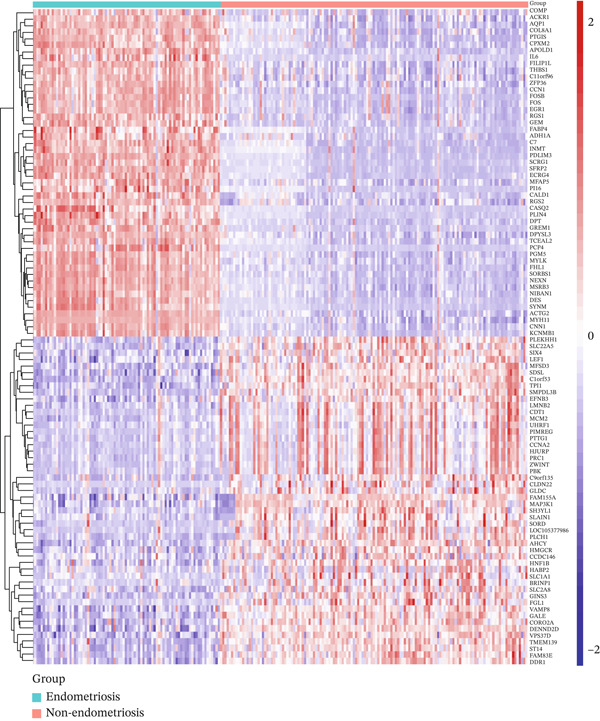
(b)

### 3.3. Functional Enrichment Analysis of DEGs

To explore the biological functions and pathways associated with the DEGs, GO and KEGG pathway enrichment analyses were performed. As shown in Figure 4a, GO analysis indicates that the DEGs were significantly enriched in BP, such as muscle tissue development, extracellular matrix organization, and actin filament‐based processes. These processes are primarily related to cellular structure, motility, and tissue remodeling. As shown in Figure 4b, KEGG pathway analysis identified multiple significantly enriched signaling pathways, including the cytokine–cytokine receptor interaction, human T‐cell leukemia virus 1 infection, and focal adhesion pathways. These pathways are closely related to immune regulation, cell adhesion, and inflammatory responses, suggesting that the gene set may play a role in the immune microenvironment and tissue integrity.

Figure 4Enrichment analysis of DEGs. (a) GO enrichment analysis. The *x*‐axis represents the number of genes enriched in the corresponding GO entry. The *y*‐axis represents biological process (BP), cellular component (CC), and molecular function (MF), respectively. (b) KEGG pathway analysis.

**Figure:**
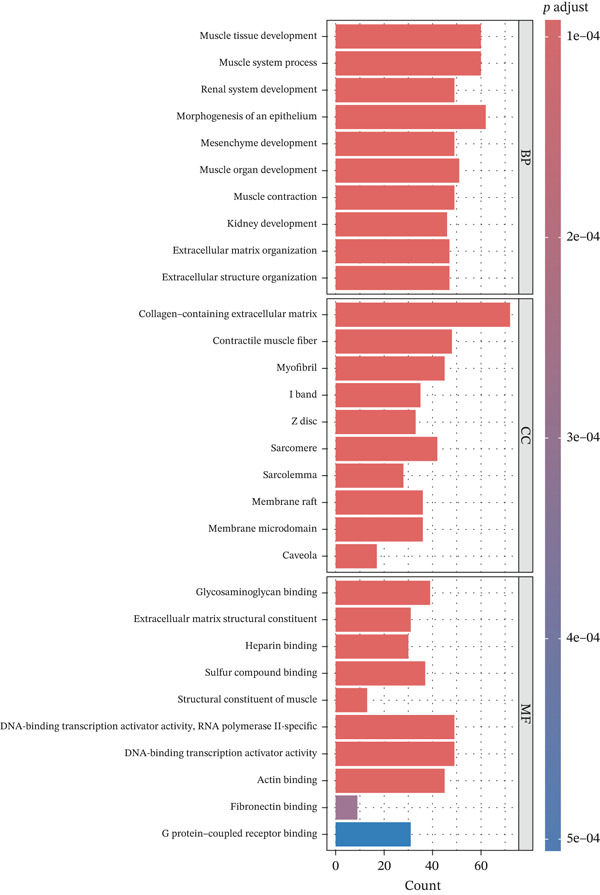
(a)

**Figure:**
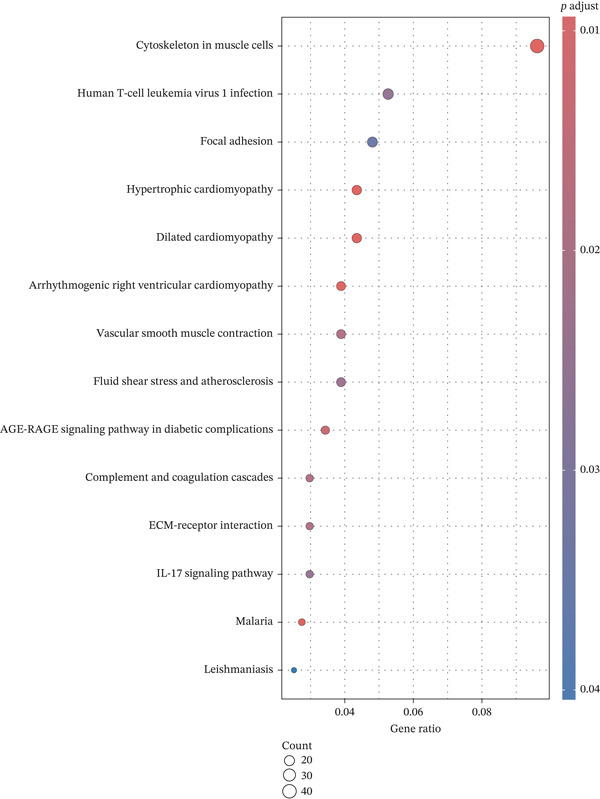
(b)

In particular, the BP function of renal system development, kidney development, metanephros development, and morphogenesis of an epithelium is related to EM. EM can involve the ureters without warning, which can lead to hydronephrosis and loss of kidney function [11]. Epithelial–mesenchymal transition (EMT) plays a key role in the formation and development of EM, and osteopontin splicing isoforms can promote endometrial epithelial cell proliferation, migration, and the process of EMT, driving the development of EM [12, 13]. Furthermore, GO enrichment in the MF category identified significant involvement of DNA‐binding transcription activator activity, particularly RNA polymerase II–specific transcription factors, which have been implicated in the transcriptional dysregulation observed in endometriotic lesions, highlighting their potential as therapeutic targets in EM pathogenesis [14, 15]. Notably, PAX8, IRX3, SOX17, SIX4, and TCF21 were commonly enriched in these GO functions, highlighting their potentially central role in driving the aberrant gene expression and tissue remodeling observed in EM.

### 3.4. Identification of EM‐Specific Genes Using RF

Model performance was evaluated using six metrics: ACC (proportion of correct predictions), precision (PRE, positive predictive value), SEN (true positive rate), SPE (true negative rate), *F*1‐score (FSC, harmonic mean of PRE and SEN), and area under the receiver operating characteristic curve (AUC).

In this study, genes specifically associated with EM were identified by machine learning models. Three widely used supervised learning algorithms were developed and optimized based on training sets, including RF, SVM, and LR. We evaluated the prediction performance of each model on the corresponding test folds using a comprehensive set of performance metrics, including ACC, PRE, SEN, FSC, SPE, and the AUC. As shown in Table 2, the RF model demonstrated excellent performance in distinguishing DiE samples and non‐DiE samples, with an ACC of 0.9821, PRE of 0.9882, FSC of 0.9767, SEN of 0.9655, and AUC of 0.9791. Although SVM achieved a slightly higher AUC of 0.9810, its SEN was lower than that of RF, indicating a weaker ability to detect positive cases. LR also showed good performance but was slightly inferior to RF in terms of ACC, PRE, and FSC. Therefore, RF was selected for subsequent analysis considering the comprehensive performance. The AUC value of RF is all greater than 0.97 in each fold (Figure 5a). A total of 343 genes with nonzero characteristic importance of RF were found at each fold, further indicating that these genes are very important. The 343 genes are EM‐specific genes (Figure 5b).

**Table 2:** The performance of RF, SVM, and LR.

	ACC	PRE	FSC	SEN	AUC (95% CI)
RF	0.9821	0.9882	0.9767	0.9655	0.9791 (0.9674–0.9908)
SVM	0.9821	0.9765	0.9765	0.9765	0.9810 (0.9698–0.9922)
LR	0.9777	0.9647	0.9704	0.9762	0.9774 (0.9651–0.9897)

Figure 5Performance of RF in identifying endometriosis‐specific genes. (a) The AUC of five‐fold of RF. (b) The Venn plot of genes with nonzero importance in each fold model.

**Figure:**
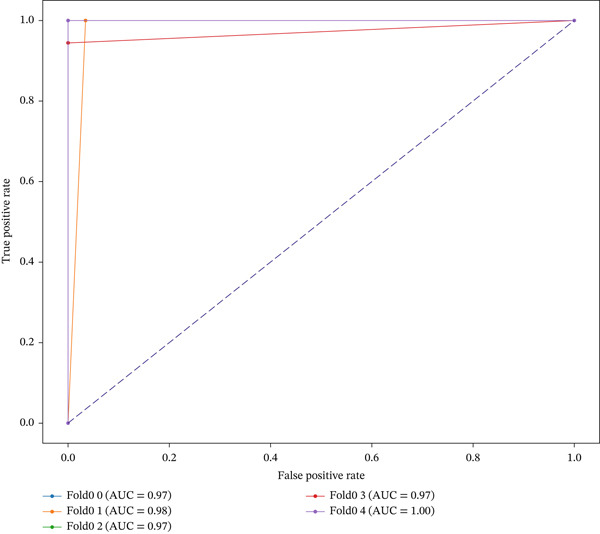
(a)

**Figure:**
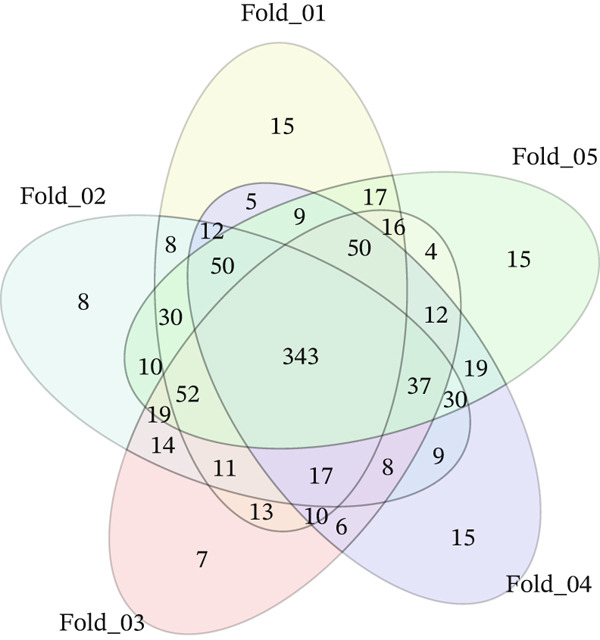
(b)

Having identified PAX8 as the most discriminative feature through machine learning algorithms, we next sought to elucidate the biological mechanisms and functional implications of PAX8 dysregulation in EM pathogenesis.

### 3.5. WGCNA Reveals a Key Gene Module Negatively Correlated With EM

To understand the biological pathways associated with PAX8 and its potential role in EM, we performed comprehensive functional enrichment analyses using GO, KEGG, and GSEA.

To explore the biological relevance of the machine learning–identified genes, we further performed WGCNA to identify disease‐associated gene modules. To elucidate the molecular mechanisms of EM, we used WGCNA to identify gene modules closely associated with the disease. Using endometrial tissue samples from EM patients and healthy controls, we constructed a WGCNA. Based on scale‐free topology criteria, we selected a soft‐thresholding power of 11, which ensured appropriate network connectivity and preserved the scale‐free network property (Figure 6a). Hierarchical clustering was then conducted based on gene expression profiles, and the resulting clustering dendrogram is shown in Figure 6b. Using a dynamic tree‐cutting algorithm and subsequent module merging, we ultimately identified three distinct coexpression modules (Figure 6c).

Figure 6(a–c) Construction and identification of key gene modules by WGCNA.

**Figure:**
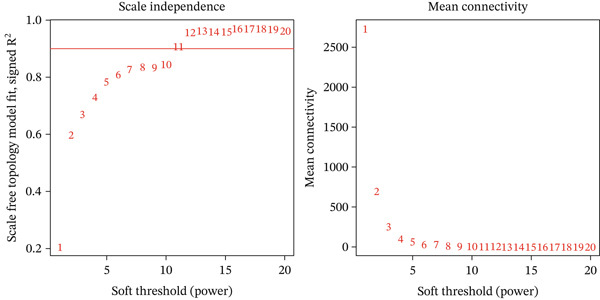
(a)

**Figure:**
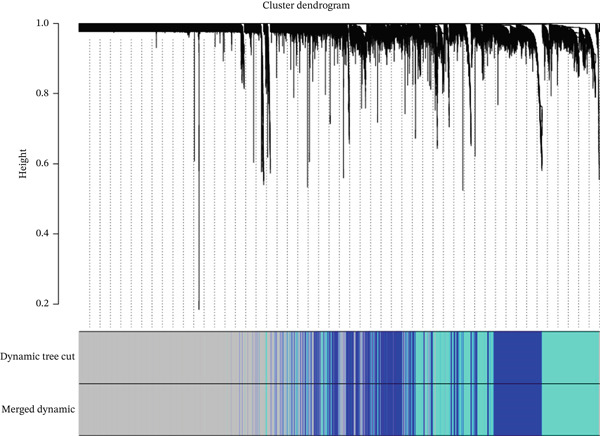
(b)

**Figure:**
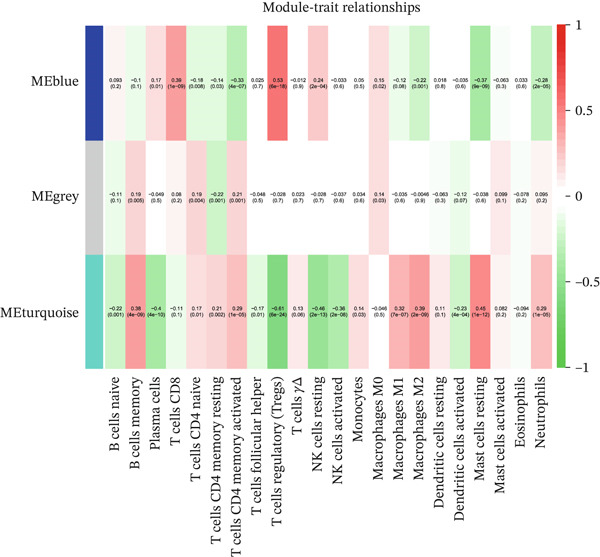
(c)

To determine the relationship between these modules and EM, we calculated the correlation between MEs and the EM phenotype. The results revealed that the MEturquoise module was significantly negatively correlated with EM (correlation coefficient cor = −0.61, *p* = 6 × 10e − 24). This module contains 5363 genes and was therefore identified as the key module most strongly associated with EM, serving as the focus for subsequent functional and mechanistic analyses.

### 3.6. Analysis of the Function of the PAX8 Gene in EM

Based on the overlap between machine learning–identified genes and WGCNA key modules, PAX8 was selected for downstream functional and immune‐related analyses. To further elucidate the role of key genes in EM, we integrated the findings from the MEturquoise identified through WGCNA and the EM‐specific genes identified by the RF model, and 307 overlapping genes were found among these genes (Figure 7c). Significantly, among the five genes identified in 3.3, PAX8 and TCF21 were recognized in these 307 genes. TCF21 has been previously reported as a therapeutic target in EM [16], which validates the reliability of our identified genes. We then focus on PAX8 for further analysis.

Figure 7Comprehensive analysis of PAX8 in endometriosis. (a) Downregulation of PAX8 in endometriosis samples. (b) Significant difference in PAX8 expression between DiE samples and non‐DiE samples. (c) Overlapping genes between the MEturquoise module and RF model. (d) Immune infiltration analysis of PAX8. (e) GSEA GO analysis of PAX8‐associated gene sets. (f) GSEA KEGG analysis of PAX8‐associated gene sets. Statistical Statistical significance is indicated as follows:  ^∗^ *p* < 0.05,  ^∗∗^ *p* < 0.01,  ^∗∗∗^ *p* < 0.001.

**Figure:**
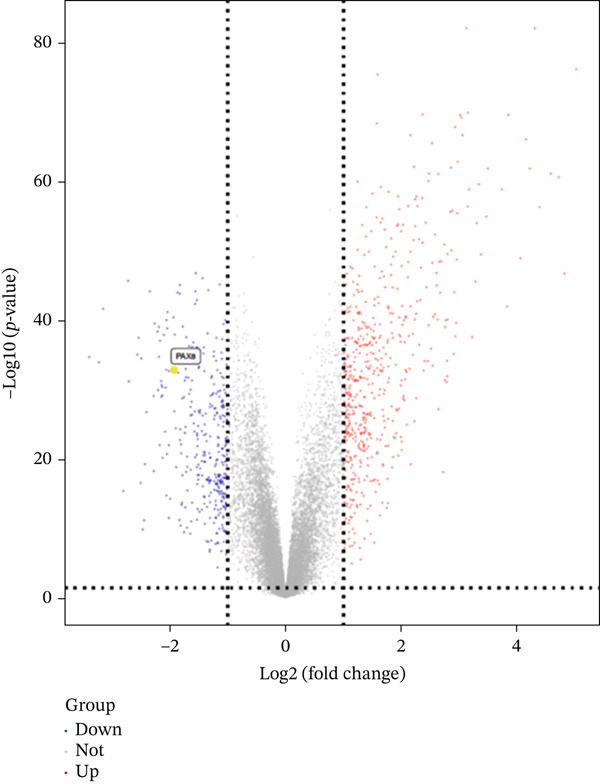
(a)

**Figure:**
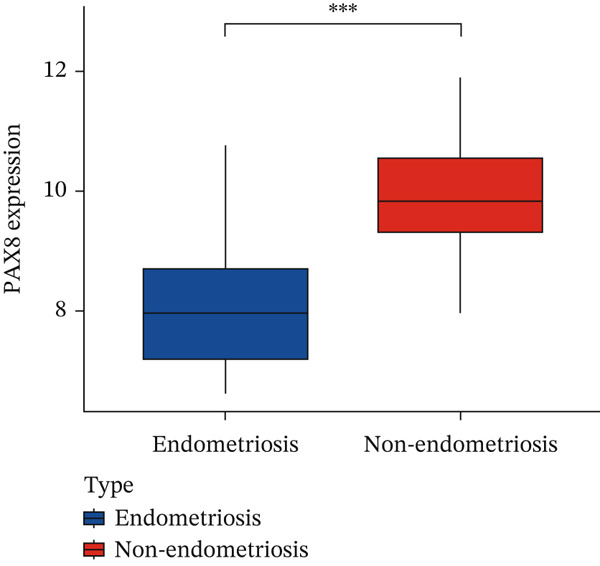
(b)

**Figure:**
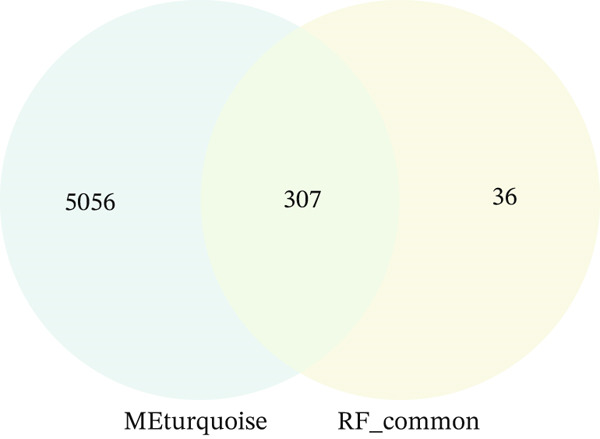
(c)

**Figure:**
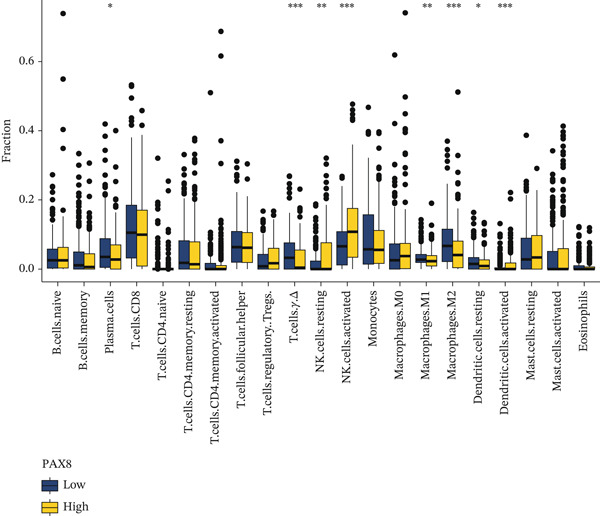
(d)

**Figure:**
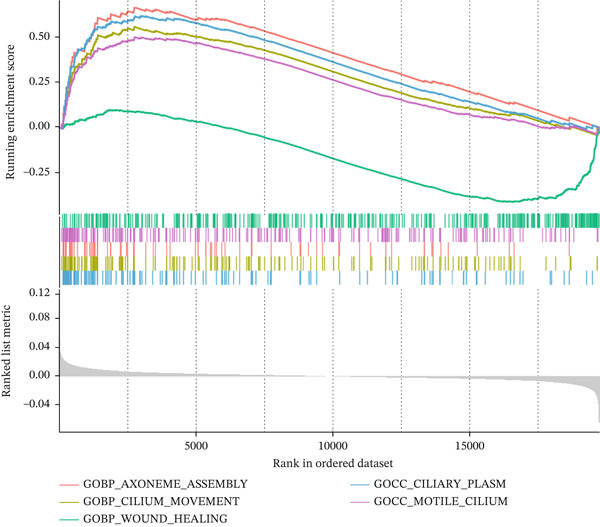
(e)

**Figure:**
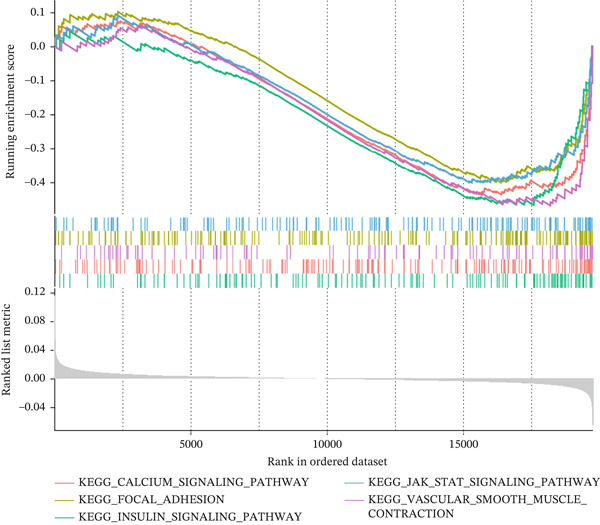
(f)

#### 3.6.1. Expression Analysis of PAX8

PAX8 is a downregulated gene in DiE samples compared to non‐DiE samples (Figure 7a). Figure 7b demonstrates a significant difference in PAX8 expression levels between DiE and non‐DiE samples, highlighting its potential as a therapeutic target for EM.

#### 3.6.2. Immune Infiltration Analysis of PAX8

The relationship between PAX8 expression and the infiltration levels of various immune cells in EM tissues was explored by immune infiltration analysis. As shown in Figure 7d, PAX8 expression was significantly correlated with infiltration levels of various immune cells, including B cells, T cells, NK cells, monocytes, macrophages, dendritic cells, and mast cells. These findings indicate that PAX8 expression is significantly correlated with the immune infiltration landscape in EM lesions. Specifically, the expression level of PAX8 was negatively correlated with the infiltration of NK cells and mast cells, suggesting that the downregulation of PAX8 may be associated with the immune dysregulation observed in EM.

#### 3.6.3. GSEA of PAX8

GSEA was applied to identify BPs and pathways significantly associated with PAX8 expression in EM. As shown in Figure 7e, GSEA identified several GO terms associated with cilia motility, axonemal assembly, and wound healing that were significantly enriched in the PAX8‐related gene set. These enriched terms suggest that PAX8 may be involved in processes related to cilia function and tissue repair mechanisms in EM.

In addition, KEGG pathway analysis (Figure 7f) highlighted several pathways that were significantly enriched in the concentration of PAX8‐related genes. These pathways include calcium signaling, focal adhesion, insulin signaling, JAK‐STAT signaling, and vascular smooth muscle contraction. The enrichment of these pathways indicates their potential involvement in the pathogenesis of EM.

## 4. Discussion

In this study, PAX8 was identified as a promising molecular biomarker for the early and noninvasive diagnosis of EM. Transcriptomic analysis of the GEO dataset GSE141549 revealed that PAX8 was significantly downregulated in ectopic endometrial tissues as compared to ectopic counterparts. Machine learning models, including RF, SVM, and LR, have consistently highlighted PAX8 as a key discriminative feature, emphasizing its potential diagnostic utility [17].

PAX8 is an integral transcription factor in Müllerian phylogeny and has been extensively studied in gynecological malignancies. Its expression in ovarian and endometrial cancers is well documented and is a reliable immunohistochemical marker for tumors of Müllerian origin [18]. Recent studies have extended its relevance to benign gynecological diseases, including EM. For example, Arakawa et al. demonstrated that PAX8 is a highly sensitive marker of extragenital EM glands, maintaining expression even under hormonal treatment [7].

The stability of PAX8 expression is independent of hormone therapy compared to traditional markers such as CD10, ER, and PR, which are affected by hormonal fluctuations [7, 19]. It is worth noting that CA125, a commonly used serum marker in clinical practice, has limited SEN and SPE, particularly in detecting early‐stage or DiE [5]. This feature makes PAX8 a reliable candidate for consistent testing in a variety of clinical situations, including patients receiving hormone therapy.

Although the precise mechanisms remain to be elucidated, the observed association between PAX8 expression and immune cell infiltration suggests a potential immunomodulatory role of PAX8 within the ectopic endometrial microenvironment. Previous studies have shown that immune cell types such as regulatory T cells (Tregs), M2 macrophages, and NK cells contribute to immune tolerance, chronic inflammation, and impaired clearance of ectopic tissue in EM (Knez, 2024 #24). PAX8, as a key transcription factor in Müllerian‐derived tissues, may influence the expression of cytokines, chemokines, or cell adhesion molecules, thereby modulating immune cell recruitment or phenotype [20, 21]. These interactions could promote an immunosuppressive niche favoring lesion persistence. Further experimental studies are needed to validate whether PAX8 directly or indirectly affects immune signaling pathways in the context of EM [22]. It should be noted that these observations are based on correlation analyses and do not imply a direct causal or mechanistic relationship.

This study represents a pioneering effort to combine transcriptomics with machine learning approaches to identify EM biomarkers. We employed multiple algorithms (RF, SVM, and LR) to analyze gene expression profiles, ensuring the robustness of the findings. RF demonstrated superior performance in distinguishing EM samples from controls, with an AUC of 0.9791 and an ACC of 0.9821, which is a novel achievement in the field. Furthermore, we integrated WGCNA and immune infiltration analysis to uncover the BPs and pathways associated with PAX8. This comprehensive approach not only highlights PAX8 as a diagnostic marker but also provides insights into its potential role in disease pathogenesis. The correlation between PAX8 expression and immune cell infiltration levels offers a novel perspective on the immunomodulatory role of PAX8 in EM.

The strengths of our study lie in its rigorous methodology and multifaceted analysis. First, the use of high‐throughput transcriptomic data from the GEO dataset GSE141549 ensures a large sample size, enhancing the statistical power of the findings. Second, the combination of differential gene expression analysis, GO and KEGG enrichment analyses, and machine learning models provides a comprehensive understanding of PAX8′s diagnostic potential. Third, the validation of PAX8′s expression stability in hormone‐treated tissues strengthens its candidacy as a reliable biomarker. Given its stable expression across diverse clinical conditions, including hormone‐treated and fibrotic tissues, PAX8 holds promise as a robust candidate for inclusion in future diagnostic assays, such as endometrial biopsy panels or circulating transcriptomic signatures. Its integration into noninvasive screening workflows could improve early detection and reduce diagnostic delays in clinical settings.

Despite these promising findings, several limitations warrant consideration. The study′s reliance on retrospective transcriptomic data necessitates validation through prospective clinical studies and immunohistochemical analyses to confirm PAX8′s diagnostic ACC and SPE. Additionally, the dataset used in this study primarily consists of peritoneal tissue samples, which may limit the generalizability of the findings to other types of endometrioses, such as ovarian EM. Furthermore, while our analysis suggests a correlation between PAX8 expression and immune cell infiltration, the exact mechanisms underlying this relationship require further investigation. Finally, due to inherent limitations of the database, the samples used in this study lack access to more detailed patient information, such as age, ethnicity, and disease stage. Future prospective multicenter studies with larger sample sizes are warranted to further validate the findings of this study.

Our study provides a robust foundation for the application of PAX8 as a noninvasive diagnostic biomarker for EM. Future research should focus on prospective validation and exploring the clinical utility of PAX8 in diverse patient populations. This work not only advances our understanding of EM pathogenesis but also offers a promising avenue for the development of novel diagnostic tools.

## 5. Conclusion

PAX8 demonstrates significant diagnostic potential and may serve as a valuable candidate biomarker for EM, warranting further validation in prospective clinical studies. The integration of transcriptomic analysis with machine learning approaches successfully identified PAX8 as a key discriminative feature. The correlation between PAX8 expression and immune cell infiltration suggests potential immunomodulatory roles that warrant further investigation. These findings provide a foundation for developing noninvasive diagnostic strategies, though clinical validation in larger cohorts is essential before translation to clinical practice.

This study identifies PAX8 as a promising diagnostic biomarker candidate for EM based on integrated transcriptomic and machine learning analyses. PAX8 showed consistent downregulation in ectopic tissues and strong diagnostic performance across models. Our findings provide initial evidence for its potential clinical utility and offer insights into the immune‐related mechanisms of EM. Future validation in prospective clinical settings is warranted.

## Funding

No funding was received for this manuscript.

## Consent

The authors have nothing to report.

## Conflicts of Interest

The authors declare no conflicts of interest.

## Data Availability

The data used and/or analyzed during the current study are available from the corresponding author.
